# Estrogen-immuno-neuromodulation disorders in menopausal depression

**DOI:** 10.1186/s12974-024-03152-1

**Published:** 2024-06-19

**Authors:** Yuling Zhang, Xiying Tan, Chaozhi Tang

**Affiliations:** 1 College of Life Science, Henan Normal University, Xinxiang, 453007 Henan China; 2 Department of Neurology, Xinxiang City First People’s Hospital, Xinxiang, 453000 Henan China

**Keywords:** Estrogen, Menopausal depression, Inflammatory cytokine, Blood-brain barrier, Neurotransmitter, BDNF, Neuroplasticity

## Abstract

A significant decrease in estrogen levels puts menopausal women at high risk for major depression, which remains difficult to cure despite its relatively clear etiology. With the discovery of abnormally elevated inflammation in menopausal depressed women, immune imbalance has become a novel focus in the study of menopausal depression. In this paper, we examined the characteristics and possible mechanisms of immune imbalance caused by decreased estrogen levels during menopause and found that estrogen deficiency disrupted immune homeostasis, especially the levels of inflammatory cytokines through the ERα/ERβ/GPER-associated NLRP3/NF-κB signaling pathways. We also analyzed the destruction of the blood-brain barrier, dysfunction of neurotransmitters, blockade of BDNF synthesis, and attenuation of neuroplasticity caused by inflammatory cytokine activity, and investigated estrogen-immuno-neuromodulation disorders in menopausal depression. Current research suggests that drugs targeting inflammatory cytokines and NLRP3/NF-κB signaling molecules are promising for restoring homeostasis of the estrogen-immuno-neuromodulation system and may play a positive role in the intervention and treatment of menopausal depression.

**Supplementary Information:**

The online version contains supplementary material available at 10.1186/s12974-024-03152-1.

## Introduction

Depression is a major cause of suicide and mortality worldwide [1, 2]. The vast majority of patients with depression show insufficient secretion of monoamine neurotransmitters, making selective serotonin reuptake inhibitors (SSRIs) the main antidepressant drugs. SSRIs maintain a high level of 5-hydroxytryptamine (5-HT) in the synaptic cleft, which can effectively alleviate depressive episodes in the short term [2, 3] but cannot repair the autonomous regulation of neurotransmitter synthesis, secretion, release and absorption by neurons [4, 5]. Long-term use of SSRIs induces neuronal dysfunction and gradually leads to drug dependence in depressed patients [5–7]; therefore, tracking the cause of neuronal dysfunction and developing corresponding treatments may be the best way to cure depression. With the deterioration of ovarian function, the secretion of estrogen in menopausal women decreases sharply, and the serious imbalance of endocrine hormone levels results in a high incidence of major depression in menopausal women [8–11]. The relatively clear etiology of menopausal depression provides promising prospects for the investigation of neuronal dysfunction.

Some early breakthroughs in menopausal depression focused on the link between estrogen and depression [12–14]. Initially, an epidemiological survey of 16,080 women aged 35–60 years led to a preliminary understanding of menopausal depression and revealed that menopausal women had significantly more severe depressive symptoms, suggesting that changes in estrogen homeostasis are related to the development of depression [12]. Further analysis confirmed that estrogen can exert antidepressant effects by regulating the level of 5-HT and the expression of 5-HT receptors (5-HTR) on presynaptic or postsynaptic membranes [13]. Subsequently, Chhibber et al. reported that decreased estrogen receptor (ER) expression reduces brain-derived neurotrophic factor (BDNF) levels in the hippocampus, thereby inhibiting the tropomyosin receptor kinase B (TrkB) signaling pathway, leading to 5-HT2A dysfunction, attenuated synaptic plasticity, and increased susceptibility to depression in menopausal women [14]. To date, abnormalities in signaling pathways caused by estrogen deficiency have attracted much attention.

Recently, significantly abnormal proportions of T-cell subsets, activation of microglia, and elevated levels of inflammatory cytokines in the central nervous system and peripheral blood were detected during the onset of menopausal depression [15–17], and these immune disorders increase the activity of the estrogen-immuno-neuromodulation system, which may be the key to resolving neuronal dysfunction in menopausal depression. However, due to the involvement of multiple ERs [18–21], immune responses and regulatory factors [19–27] and nerve signaling molecules [26–30], the disturbance of signaling pathways in this complex system remains unclear and has not been comprehensively reviewed in menopausal depression.

To this end, focusing on the decline of estrogen levels in menopausal women, we summarize the immune system imbalance and neurological impairments caused by estrogen deficiency and analyze the detailed process and possible mechanism of estrogen-immuno-neuromodulation disorders in menopausal depression, with the aim of providing scientific directions for further elucidating the pathogenesis of menopausal depression and developing novel targeted therapeutic drugs.

## Immune imbalance in menopause

### Characteristic changes in the immune system during menopause

Characteristic changes in the immune system in menopausal women are the first clue to understanding the disorder of the estrogen-immuno-neuromodulation system in menopausal depression in order to trace the relevant signaling pathways from estrogen to specific indicators of immune imbalance.

An analysis of peripheral blood lymphocyte subsets in menopausal women and reproductive women revealed that the total number of lymphocytes in the menopausal women was lower; specifically, the number of B lymphocytes and CD4^+^ T cells were significantly lower, and the ratio of CD4^+^ to CD8^+^ T cells was also significantly lower [31]. Further diagnostic findings of serological biochemical factors revealed that the serum level of interleukin (IL)-4 (a Th2 cytokine) was increased and the level of interferon (IFN)-γ (a Th1 cytokine) was decreased in menopausal women, suggesting that the cellular immune activity of the body tended to be attenuated after the decrease in estrogen levels [32].

Another important characteristic change in the immune system of menopausal women is an increased susceptibility to inflammation. Malutan et al. compared the levels of inflammatory factors in women who were fertile, perimenopausal, postmenopausal, ovariectomized, or chronically inflammatory and observed that the levels of the inflammatory factors IL-1β, IL-8 and tumor necrosis factor (TNF)-α were significantly greater in menopausal women, while the levels of the anti-inflammatory factor IL-20 were lower [33]. Patients with perimenopausal depression have increased levels of inflammation [34]. Animal models of perimenopausal depression exhibit activation of microglia and astrocytes, as well as increased neuroinflammation and nerve damage [25, 35, 36].

### Effect of estrogen on immune imbalance during menopause

Changes in the immune system in menopausal women indicate that a decrease in estrogen levels had an important impact on immune homeostasis, leading to the development of estrogen replacement therapy (ERT). Estrogens are cholesterol-derived steroid hormones with wide ranges of biological activities that mainly include estrone (E1), estradiol (17β-estradiol, E2) and estriol (E3). Among them, E2 is the main form of estrogen in the human body, and its physiological activity is considered to be the strongest [37]. Kumru et al. demonstrated that E2 could reduce the number of CD8^+^ T cells to a certain extent, restore the ratio of CD4^+^ to CD8^+^ T cells in peripheral blood to a normal level, and significantly increase the proportion of CD19^+^ B cells and the level of IFN-γ [38]. E2 also had a restorative effect on the proportion of different lymphocyte subsets in menopausal women, but the number of CD4^+^ T cells and CD20^+^ B cells was still lower than that in reproductive women, which indicates that estrogen plays an important role in regulating the immune status of menopausal women but cannot reverse the decline in immune function caused by aging [39]. Estrogen has also been found to have a positive regulatory effect on the preservation of naïve B cells, which is conducive to the occurrence of a humoral immune response. In addition, estrogen can decrease the level of the proinflammatory cytokine IL-6 in peripheral blood to a certain extent, inhibit the secretion of inflammatory cytokines by CD4^+^ T cells, and reduce the inflammatory response in menopausal women [39]. All of this evidence suggests that estrogen can repair immune imbalances and maintain immune homeostasis during menopause.

## Possible mechanisms of immune imbalance caused by estrogen deficiency

The mechanism of immune imbalance caused by estrogen deficiency has not yet been well investigated in menopausal women [32, 40], but the understanding of this process is gradually being explored in menopausal animal models and related cell lines. The effect of estrogen is known to be exerted mainly through its receptors. ERs consists of classical nuclear receptors (ERα, ERβ and their subtypes), G protein-coupled estrogen receptor (GPER), ER-X and Gaq-ER, which are specifically expressed in the cell membrane, cytoplasm and nucleus of monocytes, macrophages, dendritic cells, neutrophils, NK cells, CD4^+^ and CD8^+^ T cells, regulatory T (Treg) cells, B cells, microglia, astrocytes, and neurons, among others [18, 20, 22, 41, 42]. When activated by estrogen, different ERs can regulate gene expression directly or in conjunction with transcription factors or can also regulate cell signal transduction pathways through second messengers such as cAMP [18, 20, 22, 41]. Recent studies on the mechanism of immune imbalance caused by estrogen deficiency during menopause have focused mainly on the regulation of inflammatory signaling pathways by ERα, ERβ and GPER. Understanding these pathways is crucial for elucidating the mechanisms of immune imbalance caused by estrogen deficiency and their potential contribution to menopausal depression.

### NLRP3 signaling pathways mediated by the classical ER

**Fig. 1:**
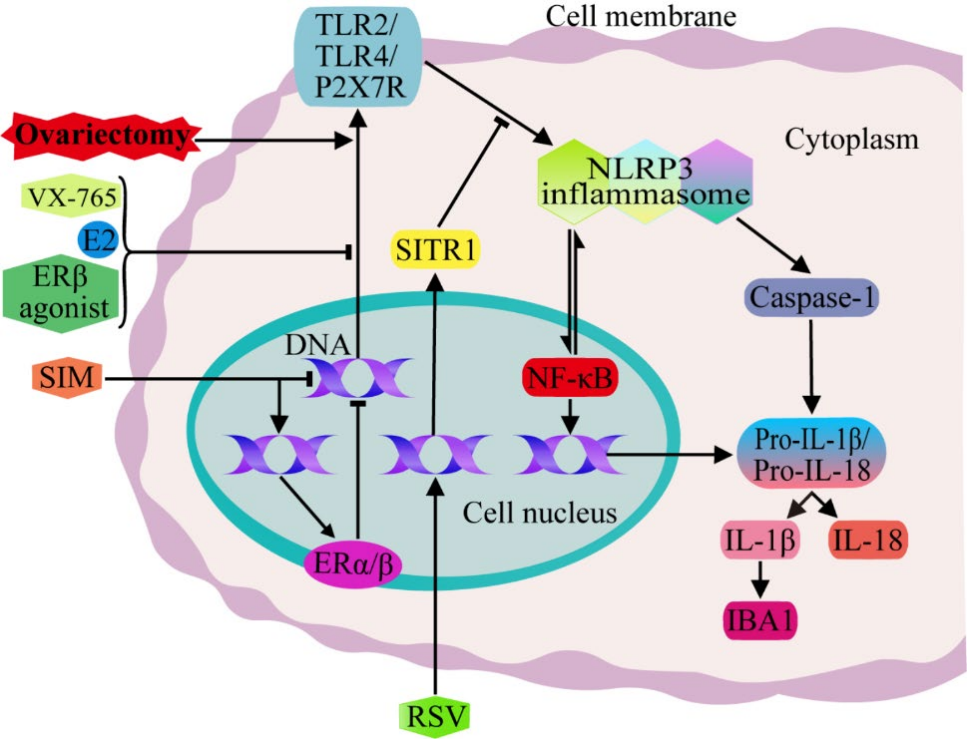
NLRP3 signaling pathways regulated by E2. Estrogen deficiency in ovariectomized mice or rats increases the expression of P2X7R, TLR2 and TLR4 in the hippocampus and further leads to the activation of NLRP3, which promotes the inflammatory cascade and increases the expression of pro-IL-1β and pro-IL-18. Moreover, NLRP3 can lead to caspase-1 activation, which in turn promotes the transformation of pro-IL-1β and pro-IL-18 into mature IL-1β and IL-18, and then, IL-1β facilitates the increase in IBA1 expression and the activation of microglia. Administration of the inflammasome inhibitors VX-765, E2 and ERβ agonists to ovariectomized mice can inhibit the expression of P2X7R, TLR2 and TLR4, block these signaling pathways, and reverse the depression and anxiety-like behavior caused by estrogen deficiency. RSV inhibits depression-like behavior by increasing the level of SIRT1 and inhibiting the activation of the NLRP3 inflammasome. SIM can also inhibit the expression of P2X7R, TLR2 and TLR4 and the activation of the NLRP3 inflammasome and its downstream signaling pathways in the hippocampus of ovariectomized rats, as well as reduce the activation of microglia induced by IL-1β, and alleviate depression in ovariectomized rats. (➝, positive regulation; ⊣, negative regulation. The meaning of these two indicators is the same across all the figures in this article.)

Nucleotide-binding oligomerization domain-like receptor protein 3 (NLRP3), the triggering component of inflammasome formation, is involved in the occurrence and treatment of menopausal depression. Xu et al. reported that estrogen deficiency increased the expression of purinergic ligand-gated ion channel 7 receptor (P2X7R), toll-like receptor (TLR) 2 and TLR4 in the hippocampus of ovariectomized mice, promoted the inflammatory cascade reaction and the formation of the NLRP3 inflammasome, stimulated nuclear factor-κB (NF-κB), increased the expression of pro-IL-1β and pro-IL-18, and finally induced depression and anxiety-like behavior in mice. Administration of the inflammasome inhibitors VX-765, E2 and ERβ agonists to ovariectomized mice blocked these signaling pathways by inhibiting P2X7R, TLR2 and TLR4 expression, thereby reversing depression and anxiety-like behaviors caused by estrogen deficiency (Fig. 1) [23]. Resveratrol (RSV), a potential replacement for E2, can inhibit the activation of NLRP3 and NF-κB in the hippocampus by increasing the levels of silent information regulator factor 2-related enzyme 1 (sirtuin 1, SIRT1), thereby restraining the increase in caspase-1 and NLRP3 inflammasome effectors caused by the activation of NLRP3, the conversion of pro-IL-1β to mature IL-1β and the strong release of IL-1β and IL-18, effectively combating depression and anxiety-like behaviors caused by estrogen deficiency (Fig. 1) [43]. Menze et al. analyzed the mechanism of simvastatin (SIM) in neuroprotection and depression-like behavior resistance and revealed that SIM can also inhibit the expression of P2X7R, TLR2 and TLR4 in the hippocampus of ovariectomized rats and block the activation of the NLRP3 inflammasome, thus decreasing the levels of the proinflammatory cytokines IL-1β and IL-18 and reducing the expression of ionized calcium-binding adapter molecule 1 (IBA1) and the activation of microglia. SIM also significantly increased the expression of ERα and ERβ in the hippocampus and the expression of ERβ in the uterus of ovariectomized mice but did not increase uterine weight, suggesting that SIM may be a safer alternative to hormone replacement therapy for the management of postmenopausal depression (Fig. 1) [44]. These studies suggest that the NLRP3 inflammasome may become a potential therapeutic target for estrogen deficiency-related affective disorders such as depression.

### NF-κB signaling pathways mediated by the classical ER

**Fig. 2:**
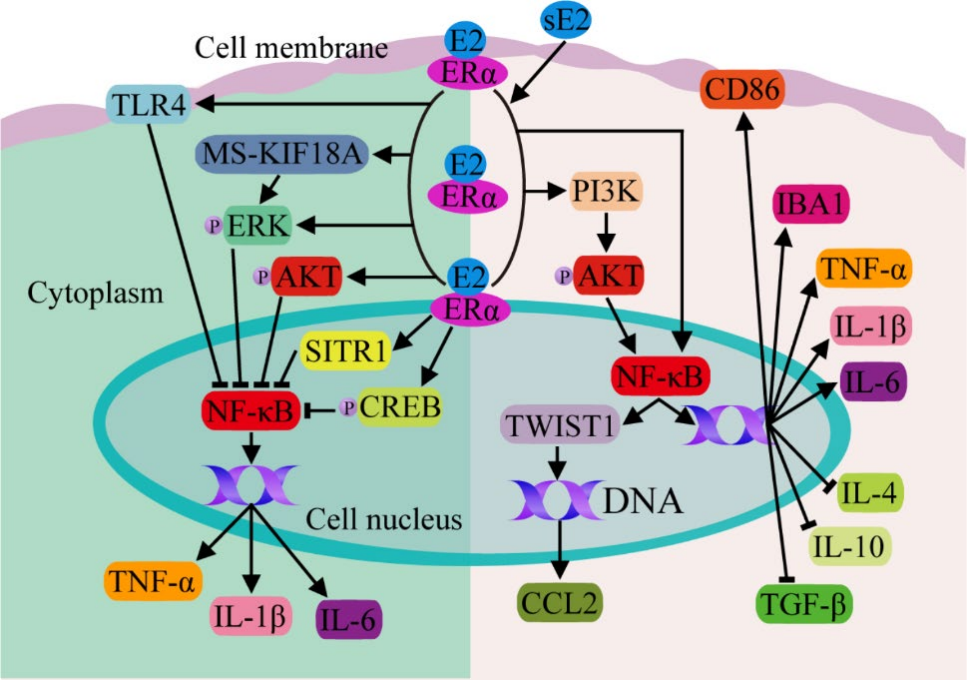
NF-κB signaling pathways mediated by classical ER. In general, upon binding to ERα in the membrane, cytoplasm or nucleus, E2 can inhibit the activation of NF-κB by regulating TLR4, MS-KIF18A, ERK, AKT, CREB, SIRT1 and other signal transduction molecules, thereby decreases the expression of the proinflammatory cytokines TNF-α, IL-1β and IL-6. However, supraphysiologic doses of E2 (sE2) abnormally activate NF-κB in microglia, resulting in increased expression of IBA1, CD86 and the proinflammatory cytokines TNF-α, IL-1β, and IL-6 and decreased expression of the anti-inflammatory cytokines TGF-β, IL-10, and IL-4. Moreover, E2 can promote the proliferation, migration and invasion of ER^+^ breast cancer cells by activating the PI3K/AKT/NF-κB/TWIST1/CCL2 signaling pathway

NF-κB, an important nuclear transcription factor, can be regulated by estrogen through multiple signaling pathways. Ovariectomy experiments in animals have demonstrated that the decreased levels of E2 activate NF-κB signaling pathway in microglia, converting microglia from the M2 subtype to the M1 subtype and resulting in the production and secretion of large amounts of inflammatory cytokines such as TNF-α, IL-1β and IL-6, which leads to cognitive impairment and depressive behavior [45, 46]. Another comparative study showed that E2 supplementation improved depression-like behavior in aging female mice by reducing NF-κB activation and the expression of the inflammatory cytokines TNF-α, IL-1β, and IL-6. Both ERα and SIRT1 antagonists can reverse the effects of E2, suggesting that E2 may protect aging female mice from depression through the E2/ERα/SIRT1/NF-κB signaling pathway [19] (Fig. 2). By implanting E2 release pellets into ovariectomized middle-aged female rats, Pratap et al. reported that E2 could decrease the level of p-NF-κB and increase the expression of the molecular markers phosphorylated extracellular signal regulated protein kinase (p-ERK), phosphorylated cAMP-responsive element binding protein (p-CREB), phosphorylated protein kinase B (p-PKB or AKT), and nitric oxide in the frontal cortex, striatum, and other brain areas [24] (Fig. 2). In addition, E2 can inhibit the activation of microglia and astrocytes and reduce the expression of proinflammatory cytokines such as TNF-α, IL-1β and IL-6 through TLR4/NF-κB signaling pathway [22], and our previous studies indicated that the occurrence and exacerbation of depression are closely related to the NF-κB-related signaling pathway in microglia [27] (Fig. 2). E2 can also activate marrow stromal kinesin family member 18 A (MS-KIF18A) in the cytoplasm after binding to ERα, and MS-KIF18A further activates the rapid phosphorylation of ERK, thereby inhibiting the activation of NF-κB [41] (Fig. 2).

The above results [19, 22, 24, 41, 45–47] support the idea that estrogen inhibits the activation of the NF-κB signaling pathway and the secretion of inflammatory cytokines in reproductive women, maintaining immune homeostasis in the body (especially in the central nervous system), while during menopause, a decrease in estrogen levels triggers the activation of NF-κB signaling pathways and the expression of inflammatory cytokines, resulting in an immune imbalance in the body, which further promotes neuronal damage and menopausal depression.

Unexpectedly, Li et al. reported that supraphysiologic doses of E2 can induce neuronal damage and aggravate the depressive behavior of ovariectomized mice by abnormally activating the expression of IBA1 and CD86 in microglia and disrupting brain glycerophospholipid metabolism through ERα/NF-κB, increasing the expression of the proinflammatory cytokines TNF-α, IL-1β, and IL-6 and decreasing the expression of the anti-inflammatory cytokines TGF-β, IL-10, and IL-4 [48] (Fig. 2). In addition, studies on the relationship between E2 and breast cancer showed that E2 can upregulate the transcription factor TWIST1 (a member of the basic helix-loop-helix transcription factor family) and NF-κB by activating the ERα/PI3K (phosphatidylinositol 3-hydroxy kinase)/AKT/NF-κB signal transduction pathway, thus positively regulating C-C motif chemokine ligand 2 (CCL2) synthesis and autocrine signaling to promote the proliferation, migration and invasion of ER^+^ breast cancer cells [49] (Fig. 2). These results suggest that the E2/ERα/NF-κB signaling pathway regulates cellular or body inflammation levels with diverse outcomes [19, 22, 24, 41, 47–49]. Whether the reason for this phenomenon is related to the dose of E2 or the different ways in which E2 regulates the central nervous system and peripheral immune system requires further precise investigation.

### Inflammatory signaling pathways mediated by GPER

**Fig. 3:**
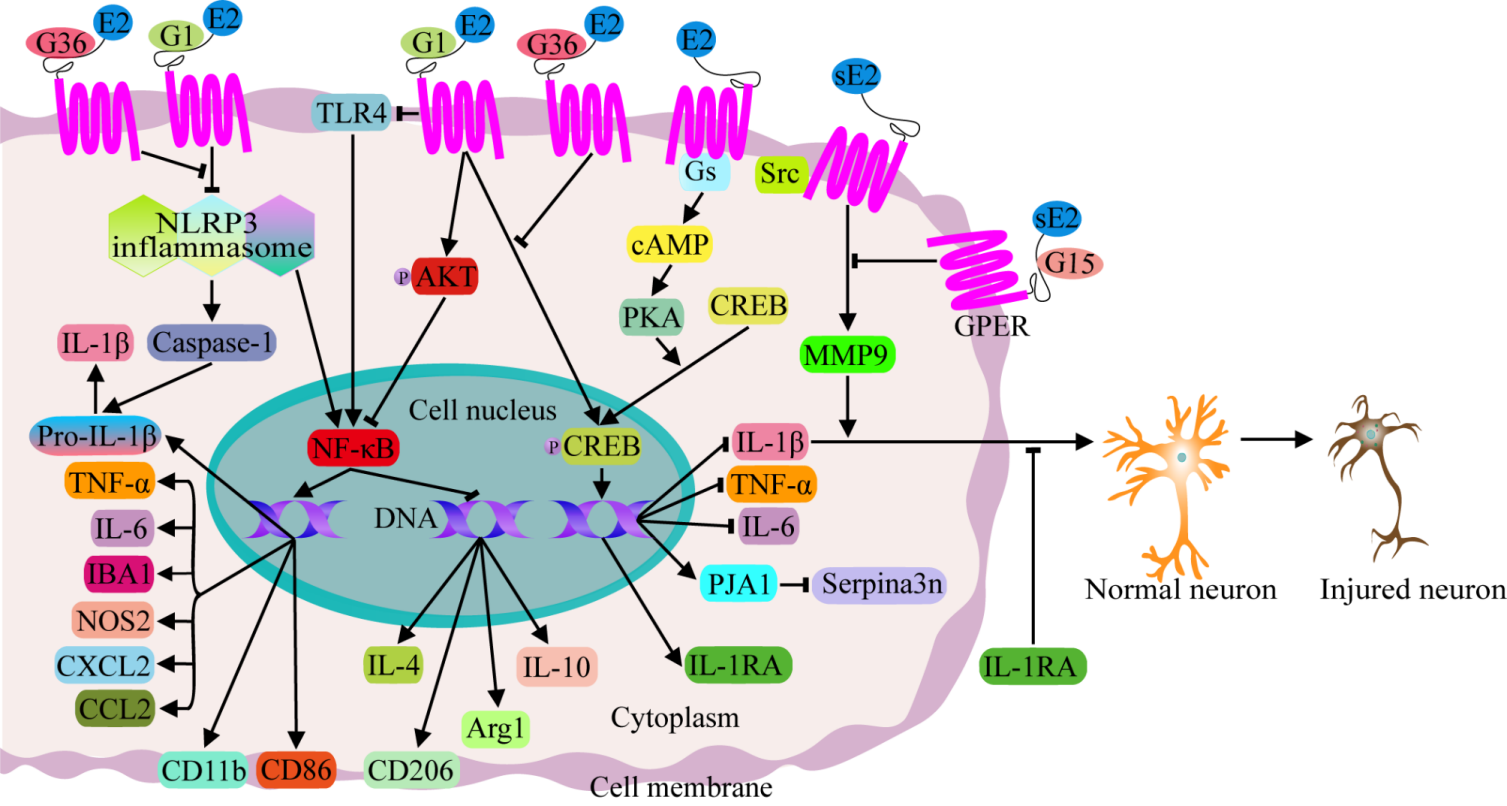
Inflammatory signaling pathways mediated by GPER. E2 can inhibit the expression of TNF-α and IL-1β through GPER, and the GPER agonist G1 can promote the anti-inflammatory effect of E2. G1 can also reduce the expression of IL-6, CD86, CD11b, MCP-1, IBA1, NOS2, and MIP-2, as well as the activation of NF-κB, and increase the expression of the anti-inflammatory factors IL-4, IL-10, Arg1 and CD206. The mechanism of the GPER agonist G1 is closely related to the phosphorylation of AKT and the inhibition of TLR4. GPER activation can also reduce NLRP3 inflammasome formation, caspase-1 activation mediated by the NLRP3 inflammasome and downstream pro-IL-1β transformation into IL-1β, as well as downstream NF-κB signaling, and upregulate the expression of IL-1RA mediated by p-CREB (IL-1RA enhances the defense of neurons against IL-1β). The GPER agonist G1 enhances this anti-inflammatory effect of GPER, while the GPER antagonist G36 reverses this effect. Additionally, GPER can decrease the expression of TNF-α, IL-6 and IL-1β, positively regulate PJA1 expression, and decrease the accumulation of Serpina3n through the Gs/cAMP/PKA/CREB signaling pathway. A too high level of E2 can also lead to the overexpression of GPER, Src and MMP-9 in neurons and subsequently activate IL-1β to result in harmful effects on the nervous system. The GPER antagonist G15 can reverse the damage caused by E2-induced GPER overactivation

G protein-coupled receptor (GPR) 30 is a new type of ER, also known as GPER, which is widely distributed in the central nervous system (hippocampus, cortex, hypothalamus, striatum and brain stem) and is associated with emotional regulation [50–54]. Estrogen can not only regulates the NLRP3 and NF-κB signaling pathways through ERα and ERβ but also regulates inflammation levels, microglial polarization and astrocyte activity in the central nervous system through GPER-related signaling pathways to maintain immune homeostasis in the brain and rescue brain injury, hypomnesia and cognitive impairment [20, 21, 52, 54–56] (Fig. 3).

Zhao et al. demonstrated that E2 can inhibit the expression of inflammatory cytokines in microglia through GPER, and the GPER agonist G1 can promote the anti-inflammatory effect of E2, but GPER antagonist G15 or GPER knockdown can eliminate the anti-inflammatory effect of E2 [21]. In addition to inhibiting the production of TNF-α, IL-1β and IL-6 by microglia, the GPER agonist G1 can also reduce the expression of CD86, CD11b, macrophage cationic peptide 1 (MCP-1, also known as CCL2), IBA1, nitric oxide synthase 2 (NOS2), macrophage inflammatory protein 2 (MIP-2, also known as CXCL2), and NF-κB and increase the expression of the anti-inflammatory factors IL-4, IL-10, arginase 1 (Arg1) and CD206 in microglia, polarize microglia to the M2 phenotype, and rescue the damage of inflammatory factors to neurons [52, 55, 56]. The mechanism of the GPER agonist G1 is closely related to the phosphorylation of AKT and the inhibition of TLR4 [24, 52, 55, 57]. GPER activation can also reduce microglial activation, NLRP3 inflammasome formation, caspase-1 activation mediated by the NLRP3 inflammasome and downstream IL-1β production and signal transduction, as well as downstream NF-κB signaling, and strongly upregulate the expression of the anti-inflammatory factor IL-1RA (an endogenous IL-1 receptor antagonist) mediated by p-CREB (a transcription factor known to enhance the expression of IL-1RA) in hippocampal CA1 neurons. The GPER agonist G1 enhances this anti-inflammatory effect of GPER, while the GPER antagonist G36 reverses this effect [54]. The loss of GPER in astrocytes can lead to the transformation of astrocytes to the A1 phenotype, increase the expression of TNF-α, IL-6 and IL-1β, increase the accumulation of Serpina3n in the cells, damage the function of neurons, and disrupt learning and memory in female mice. Serpina3n is a molecular marker of neuroinflammation in astrocytes. Praja-1 (PJA1) mediated the effects of astrocytic GPER on learning and memory by binding to Serpina3n. GPER can positively regulate PJA1 expression by stimulating the G protein (Gs)/cyclic adenosine monophosphate (cAMP)/protein kinase A (PKA)/CREB signaling pathway in cultured rat and human astrocytes [20]. In exosomes isolated from the plasma of postmenopausal women, the levels of GPER and PJA1 mRNA were decreased, while the level of Serpina3n in plasma was increased [20]. These results indicate that the astrocyte GPER can regulate learning and memory through the GPER/PJA1/Serpina3n signaling pathway in female mice. Although E2 beneficially regulates inflammation in the central nervous system and exerts neuronal protection by binding to its membrane receptor GPER [20, 21, 52, 54–56], too high of a level of E2 can also lead to the overexpression of GPER, sarcoma protein (Src) and matrix metalloproteinase 9 (MMP-9) in neurons and subsequently activate IL-1β to result in harmful effects on the nervous system. The GPER antagonist G15 can reverse the damage caused by E2-induced GPER overactivation [58] (Fig. 3). Considering the positive regulatory effects of GPER on neuroinflammation, microglial and astrocyte activation, neuronal damage, and cognitive and memory impairment, GPER may be a potential therapeutic target for menopausal depression.

As a cell membrane receptor, GPER is more responsive to exogenous estrogen agonist therapeutic drugs, making its therapeutic mechanism is relatively easier to address and elucidate [59]. Additionally, GPER expression is notably significant in brain tissues such as the hippocampus [60, 61]. These factors suggest that GPER-based antidepressants may be more acceptable to clinical patients. However, this does not imply that ERα and ERβ are poor targets. Although the majority of classical estrogen receptors (ERs) are expressed in the nucleus, a small number of these classical ER subtypes are expressed on the cell membrane. Moreover, the activation of classical ERs can regulate gene expression more briefly and rapidly. Therefore, further analysis of the differences in the expression of various ERs in key brain regions associated with depression and the affinity preferences of estrogen alternative therapeutic drugs for these ERs will help determine the most appropriate ER target for developing antidepressant therapies. In any case, therapeutic drugs targeting key molecules such as inflammatory cytokines within the estrogen-immune modulatory signaling pathway should play an effective role in treating menopausal depression.

## Neurological impairments caused by immune imbalance

As discussed above, due to decreased estrogen levels, menopausal depression is characterized by an immune imbalance that includes abnormal activity of immune factors such as TNF-α, IL-1β, IL-6, IL-10, IL-17, IL-33, and IFN-γ. In this section, we will further focus on neurological impairments caused by immune imbalance and the detailed pathogenesis of these impairments, providing a comprehensive overview of this complex phenomenon based on the findings of menopausal depression and its related fields.

### BBB destruction

The blood-brain barrier (BBB) refers to the isolating material between brain cells or cerebrospinal fluid (CSF) and plasma and is mainly composed of capillary endothelial cells with tight junctions, the endothelial basement membrane and the astrocyte foot plate. Under normal conditions, the BBB controls the selective permeability between the components of the plasma and brain tissue and plays a protective role in brain tissue. Under aging or pathological conditions, the integrity of the BBB structure changes, and peripheral substances enter brain tissue, leading to dysfunction of brain cells or CSF.

**Fig. 4:**
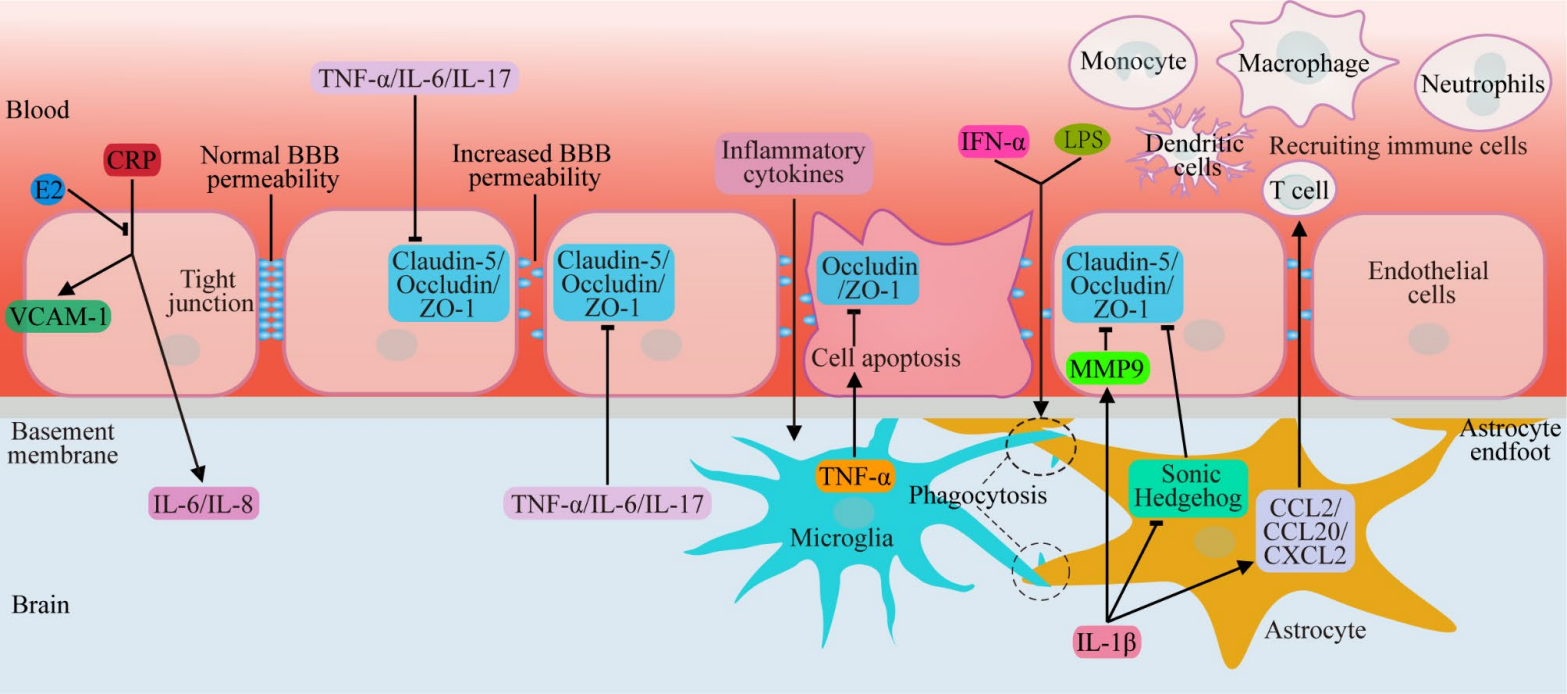
BBB destruction was triggered by immune imbalance during menopause. During menopause, a decrease in estrogen levels causes an increase in inflammatory cytokines (TNF-α/IL-6/IL-17) in the peripheral blood, which gradually decreases expression of claudin-5, ZO-1 and occludin by activating the NF-κB signaling pathway and downregulating MMP-9 expression in endothelial cells, thereby increasing the permeability of the BBB. Subsequently, inflammatory cytokines penetrate the BBB to activate microglia, which in turn secrete more inflammatory cytokines and chemokines (CCL2/CCL20/CXCL2) and recruit more aggressive immune cells, further inducing necrotic apoptosis of endothelial cells, inhibiting hedgehog protein secretion (which can improve the expression of tight junction proteins in endothelial cells) in astrocytes and triggering the phagocytosis of astrocyte footplates, thereby severely exacerbating BBB destruction. CRP can induce endothelial cell activation, and activated endothelial cells can produce more CRP, IL-6, IL-8, and VCAM-1, which further aggravate vascular endothelial cell injury. E2 pretreatment can reduce the production of the above inflammatory factors and promote vascular repair

Studies on changes in BBB structure after a decrease in estrogen levels have shown that the expression of the tight junction protein claudin-5 decreases during menopause and that the permeability of paracellular junctions to sucrose increases, thereby increasing the probability of brain edema and stroke [62, 63]. In the brain tissue of menopausal women or animal models, a decrease in estrogen levels caused an increase in the number of M1 microglia and activated astrocytes, which can secrete a large number of inflammatory cytokines. Therefore, inflammatory cytokines may play an important role in changes in BBB permeability [25, 64–74]. Several subsequent studies have demonstrated that TNF-α, IL-6 and IL-17 can reduce the expression of claudin-5, zona occludens-1 (ZO-1) and other tight junction proteins by activating NF-κB signaling pathways in endothelial cells, destroying the structure of the BBB and promoting depressive behavior [69, 70]. A large amount of TNF-α can also induce necrotic apoptosis of endothelial cells, directly leading to an increase in BBB permeability [71]. Stress-induced depression in humans is accompanied by a decrease in claudin-5 expression and an increase in IL-6 expression. Stress triggers BBB disruption, allowing IL-6 to penetrate the brain and increases the risk of depression in humans [68]. IL-1β can degrade the tight junction proteins occludin and ZO-1 by enhancing the expression of MMP-9 in endothelial cells, leading to the destruction of BBB integrity [72]. Moreover, IL-1β can inhibit the production of hedgehog proteins by astrocytes, thereby eliminating the protective effect of astrocytes on BBB integrity by downregulating the expression of tight junction proteins. IL-1β can also increase the levels of the proinflammatory chemokines CCL2, CCL20 and CXC-chemokine ligand 2 (CXCL2) in astrocytes, which further exacerbates BBB destruction and neuroinflammation after immune cell recruitment [73]. In addition, during persistent inflammation, active microglia engulf astrocyte terminals, which directly results in a loss of BBB integrity [74] (Fig. 4). Our previous studies have shown that the expression of C-reactive protein (CRP) is increased in patients with poststroke depression [75]. Cossette et al. reported that CRP can induce endothelial cell activation and that activated endothelial cells can produce more inflammatory factors, such as CRP, IL-6, IL-8 and vascular cell adhesion molecule-1 (VCAM-1), which further aggravate vascular endothelial cell injury. E2 pretreatment can reduce the production of the above inflammatory factors and promote vascular repair [76].

Based on the above findings, we hypothesize that some peripheral inflammatory cytokines first attack vascular epithelial cells and decrease the expression of tight junction proteins, leading to an increase in BBB permeability. Then, these inflammatory cytokines penetrate the BBB to activate microglia, which in turn secrete more inflammatory cytokines and chemokines and, by inducing necrotic apoptosis of endothelial cells and triggering phagocytosis of the astrocyte footplates, severely exacerbate the destruction of the BBB. Ultimately, this process leads to neuronal damage and menopausal depression.

### Neurotransmitter dysfunction

Neurotransmitters in the central nervous system of patients with major depression, including 5-HT [77, 78], dopamine (DA) [78], norepinephrine (NE) [78], γ-aminobutyric acid (GABA) [79] and glutamate (Glu) [79] have received increasing amounts of attention. We next outline the effects of inflammatory cytokines on neurotransmitter dysfunction and the associated pathogenesis in order to enhance the understanding of neurological impairments caused by immune imbalance.

#### 5-HT

5-HT, also called serotonin, is an indoleamine neurotransmitter in brain tissue that can regulate pleasant emotions. TNF-α, IL-1β and IL-6 promote depression and inhibit the functional activity of serotonergic neurons.

On the one hand, TNF-α, IL-1β and IL-6 can activate indoleamine 2,3-dioxygenase (IDO), tryptophan 2,3-dioxygenase (TDO) and monoamine oxidase (MAO), which degrade tryptophan (Trp). IDO and TDO catalyze the oxidation of Trp to initiate the metabolic cascade, leading to Trp entering the kynurenine (Kyn) metabolic pathway and no longer synthesizing 5-HT [80–82]. MAO catalyzes the degradation of 5-HT to 5-hydroxyindoleacetic acid (5-HIAA). 5-HIAA levels are significantly associated with depression and anxiety scores in patients with somatic symptom disorder, and elevated IL-6 levels exhibit mediating effects [83].

**Fig. 5:**
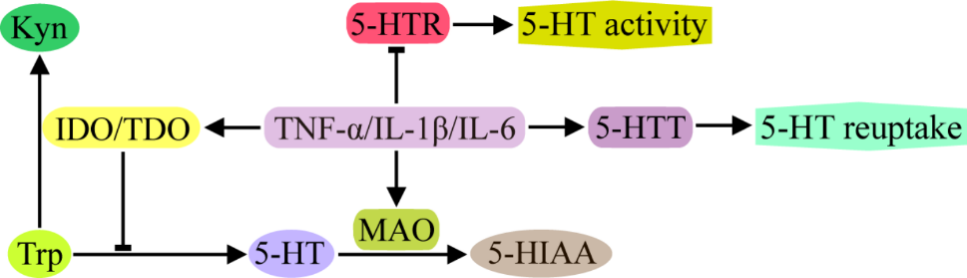
Effects of inflammatory cytokines on 5-HT synthesis, degradation, reuptake and 5-HTR expression. TNF-α/IL-1β/IL-6 activate IDO and TDO to catalyze Trp to enter the Kyn metabolic pathway, reduce 5-HT synthesis, stimulate MAO to promote 5-HT degradation into 5-HIAA, increase the expression of 5-HTT in the presynaptic membrane, aggravate 5-HT reuptake in the synaptic cleft, inhibit 5-HTR expression in the postsynaptic membrane and attenuate 5-HT efficacy

On the other hand, TNF-α, IL-1β and IL-6 can increase the expression of 5-HT transporters (5-HTT or SERT) in the presynaptic membrane of neurons and promote 5-HT transport from the synaptic cleft to the inner cell body of neurons, leading to a reduction in 5-HT in the synaptic cleft and subsequent weakening of the excitatory potential at the receptor [82, 84]. In addition, inflammatory cytokines also inhibit the expression of 5-HTR, thereby blocking or attenuating the functional activity of serotonergic neurons [85] (Fig. 5).

#### DA

DA is a catecholamine neurotransmitter in the brain that induces pleasure. Studies have shown that increased levels of TNF-α and IL-1β in CSF are positively correlated with decreased levels of DA and the severity of dopaminergic neuron damage [86, 87]. The influence of inflammatory cytokines on DA involves synthesis, packaging, metabolism, release and reuptake.

**Fig. 6:**
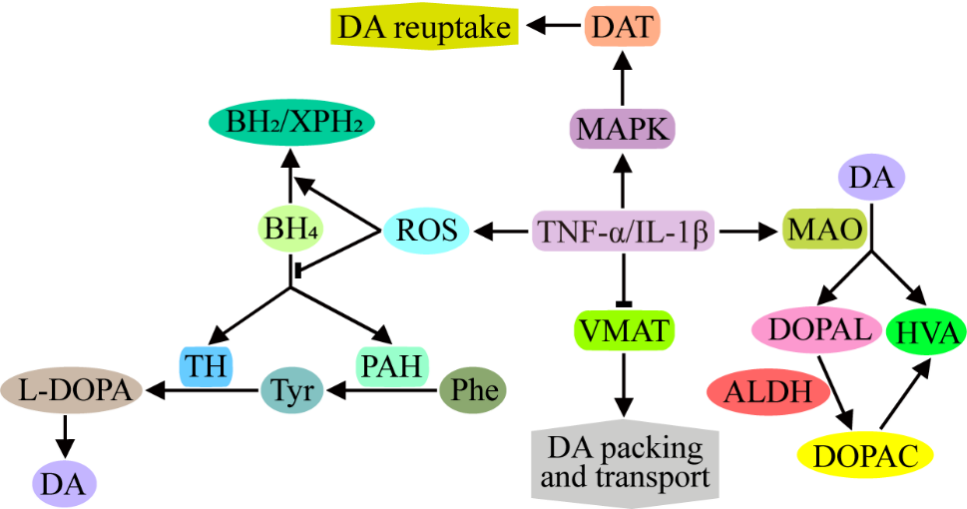
Effects of inflammatory cytokines on DA synthesis, packaging, transport, degradation and reuptake. TNF-α and IL-1β can increase ROS levels and subsequently promote the oxidation of BH_4_ to XPH_2_ and BH_2_, which further leads to the inactivation of TH and PAH and a reduction in DA synthesis. TNF-α and IL-1β are also thought to interfere with DA packaging and transport by reducing VMAT expression, and unpackaged DA is easily degraded to DOPAL and HVA by MAO. Furthermore, DOPAL is oxidized by ALDH to DOPAC, resulting in the massive metabolism of the synthesized DA. In addition, TNF-α and IL-1β can activate DAT through the MAPK signaling pathway, increasing the reuptake of DA

DA synthesis depends on tyrosine hydroxylase (TH), the rate-limiting enzyme that converts tyrosine (Tyr) to levodopa (L-DOPA). Tyr is derived from phenylalanine (Phe) via phenylalanine hydroxylase (PAH), and TH and PAH require the common cofactor tetrahydrobiopterin (BH_4_). However, high levels of TNF-α and IL-1β can cause an increase in intracellular reactive oxide species (ROS), which promotes the oxidation of BH_4_ to dihydroxanthopterin (XPH_2_) and dihydrobiopterin (BH_2_), thereby blocking TH and PAH activity and inhibiting DA synthesis [88] (Fig. 6). E2 treatment can not only decrease the expression of TNF-α, IL-1β and IL-6 but also improve the production of BH_4_ [89].

TNF-α and IL-1β are also thought to reduce the expression of vesicle monoamine transporter (VMAT), which are not conducive to DA packaging and transport [90, 91] (Fig. 6). Unpackaged DA can be degraded to 3,4-dihydroxyphenzene acetaldehyde (DOPAL) by MAO on the mitochondrial membrane; the latter is further oxidized by aldehyde dehydrogenase (ALDH) to 3,4-dihydroxyphenylacetic acid (DOPAC), resulting in the massive metabolism of the synthesized DA, which cannot be released from the presynaptic membrane [92].

In addition, TNF-α can increase the degradation of DA by extracellular MAO and produce a large amount of the metabolite homovanillic acid (HVA), which is not conducive to the activation of receptors on the postsynaptic membrane by high concentrations of DA [93] (Fig. 6).

Other studies have shown that TNF-α and IL-1β can activate 5-HTT and DA transporter (DAT) through the receptor-coupled mitogen-activated protein kinase (MAPK) signaling pathway, thereby increasing the reuptake of 5-HT and DA by presynaptic membranes, reducing the transmitter effect, and triggering depressive behaviors [94–97] (Fig. 6).

#### NE

**Fig. 7:**
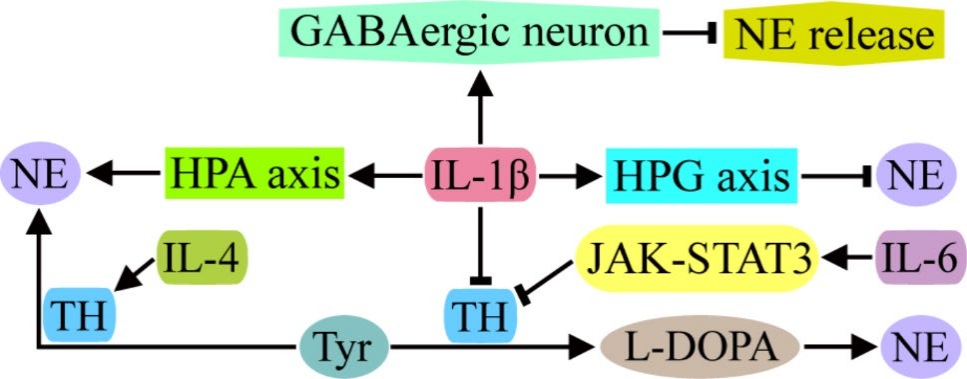
Effects of immune cytokines on NE synthesis and release. IL-1β can reduce NE synthesis by inhibiting TH activity and regulating the HPG axis, increase NE synthesis by regulating the HPA axis, and reduce NE release by activating GABAergic neurons. IL-6 can also reduce NE synthesis by inhibiting TH activity via the JAK-STAT3 signaling pathway, but the anti-inflammatory cytokine IL-4 can activate TH activity and increase NE synthesis

NE is a catecholamine neurotransmitter synthesized and secreted by sympathetic postganglionic neurons and norepinephrine neurons in the brain and is involved in the dual regulation of the hypothalamic pituitary adrenal (HPA) axis and hypothalamic pituitary gonadal (HPG) axis. NE is closely related to anxiety and sex hormone secretion. Studies on the emotional centers and areas of the brain that regulate sex hormone production have shown that IL-1β can increase NE levels in the central amygdala (CeA), bed nucleus of the stria terminalis (BNST) and paraventricular nucleus (PVN) of the HPA axis but decrease NE levels in the diagonal band of Broca (DBB), organum vasculosum lamina terminalis (OVLT), medial preoptic area (MPA), suprachiasmatic nucleus (SCN) and arcuate nucleus (Arc) of the HPG axis [98] (Fig. 7). IL-1β differentially regulates NE in different brain regions to promote the body’s adaptation to systemic immune challenges.

The mechanism by which IL-1β affects NE has been analyzed mainly through its synthesis and release. L-DOPA is a precursor substance for NE synthesis, and the supply of L-DOPA can reverse the effect of IL-1β on NE in several brain regions of rats. These results suggest that TH, the rate-limiting enzyme of L-DOPA synthesis, may be the key target of IL-1β in regulating NE synthesis [99, 100]. Similar findings have been observed for IL-6. IL-6 inhibits TH expression by activating Janus kinase-signal transducer and activator of transcription (JAK-STAT) 3 and reduces NE synthesis [101]. The anti-inflammatory cytokine IL-4 can upregulate the expression of TH and tryptophan hydroxylase (TPH), thereby reversing the negative regulatory effect of inflammatory cytokines on neurotransmitters such as NE and 5-HT and alleviating depressive behavior in rats [102]. The release of NE is regulated by a variety of transmitters. The decrease in NE in the MPA region caused by IL-1β is mainly the result of its activation of GABAergic neurons, which further exerts inhibitory effects on adrenergic neurons [103] (Fig. 7).

The effects of proinflammatory cytokines on the function of NE also include the regulation of its receptors. The binding of NE to β2-adrenoceptors on the surface of astrocytes induces the production of a large number of nerve growth factors and promotes the growth of neuronal axons, while IFN-γ can activate the MAPK signaling pathway, inhibit the expression of β2-adrenoceptors in astrocytes and attenuate the regeneration and repair of axonal branches induced by NE [104, 105].

#### GABA

GABA is an inhibitory neurotransmitter widely distributed in the brain and spinal cord, and it mainly exists in interneurons that send out negative feedback signals, thereby inhibiting 5-HT, DA and other neurotransmitters, and regulating hormone secretion, muscle contractions, blood pressure, heart rate, feeding, sleep, mood and other physiological activities. Normally, the inhibitory effect of GABAergic neurons helps coordinate the body’s homeostasis, but abnormal GABA activity can also lead to a variety of physiological dysfunctions [106].

**Fig. 8:**
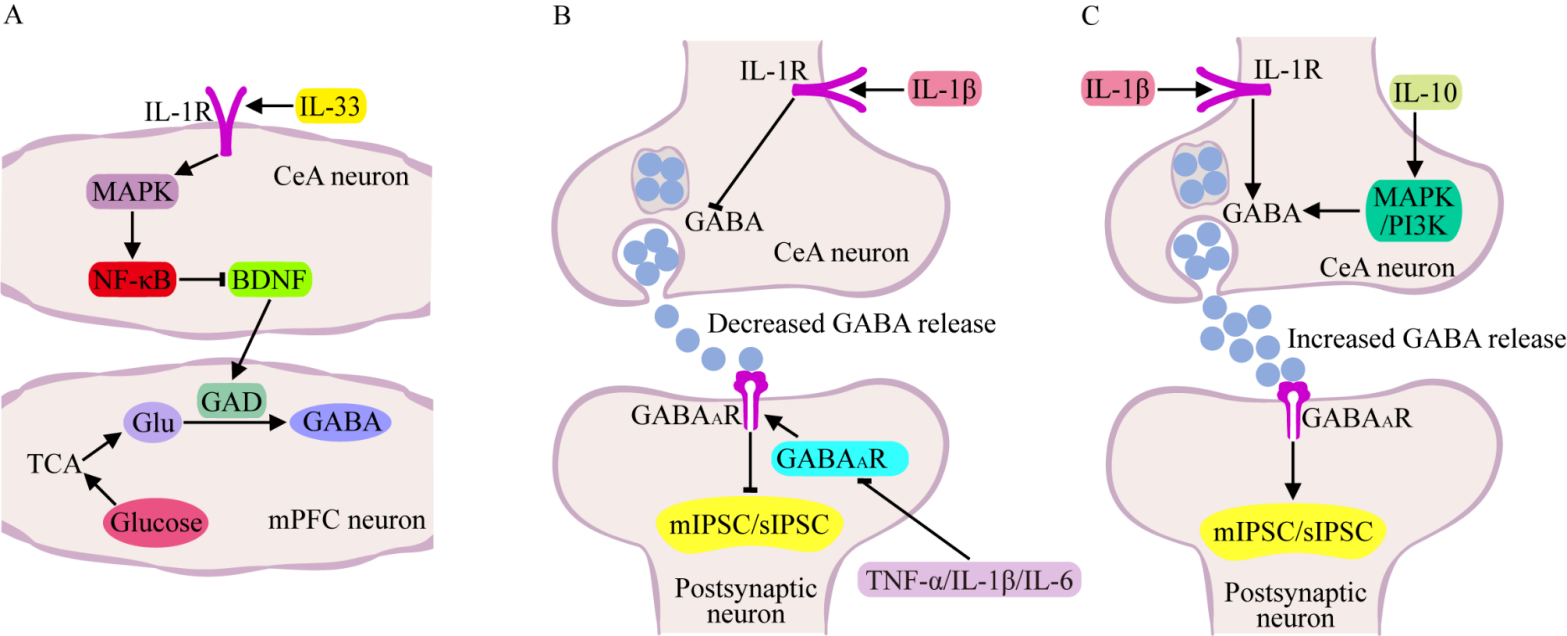
Effects of inflammatory cytokines on GABA synthesis (**A**) and release (**B**, **C**). (**A**) GABA is primarily synthesized by Glu, and GAD is the main rate-limiting enzyme for GABA synthesis. The binding of IL-33 to IL-1R first activates MAPK and further activates the NF-κB signaling pathway to inhibit BDNF synthesis, leading to a decrease in GAD expression and GABA synthesis. (**B**, **C**) IL-1β can regulate different GABAergic neurons, resulting in decreased or increased GABA release and corresponding decreases or increases in the number of IPSCs. TNF-α/IL-1β/IL-6 can also decrease the expression of postsynaptic GABA_A_R and attenuate GABA-induced IPSC. However, IL-10 promotes GABA release from GABAergic neurons and enhances IPSC through the MAPK/PI3K signaling pathway

GABA is primarily synthesized through the decarboxylation of glutamate, a process catalyzed by glutamic acid decarboxylase (GAD), which is the main rate-limiting enzyme for GABA synthesis. After synthesized GABA is released into the synaptic cleft, it first interacts with receptors on the postsynaptic membrane before being removed from the synaptic cleft by diffusion effect and actively taken up by glial cells. The ingested GABA is transformed into Glu through transamination, and the latter is further transformed into glutamine (Gln, an amide of glutamate) under the catalysis of glutamine synthetase (GS). As Gln regurgitates to neurons, it is transformed again into Glu by glutaminase (Glut), which is subsequently transformed into new GABA under the action of GAD. Studies have shown that IL-33 has a negative regulatory effect on GABA synthesis. LPS treatment of mice can lead to the overexpression of IL-33 in amygdala astrocytes. After being captured by IL-1Rs on neurons in the CeA, IL-33 can activate NF-κB and downregulate the expression of BDNF directly or indirectly through the MAPK signaling pathway, which further decreases the GAD expression region and GABA synthesis in the medial prefrontal cortex (mPFC), resulting in an attenuated inhibitory effect on GABAergic neuronal circuitry between the mPFC and CeA, and ultimately leading to anxiety-like behaviors related to excitatory neurotoxicity in the mice [29, 107] (Fig. 8A).

IL-1β, another ligand of IL-1R, can regulate GABA release in the CeA. The frequency and amplitude of miniature inhibitory postsynaptic currents (mIPSCs) in GABAergic neurons change regularly with increasing or decreasing IL-1β levels, indicating that IL-1β has a bidirectional regulatory effect on GABA release from the presynaptic membrane. This forward or reverse regulatory effect is different in various clusters of GABAergic neurons in the CeA region [108, 109]. An endogenous antagonist of IL-1β can competitively bind to IL-1R but generally does not cause downstream signals. However, its regulatory effect on spontaneous inhibitory postsynaptic currents (sIPSCs) of GABAergic neurons in the CeA region was opposite to that of IL-1β. These results indicate that IL-1β can also indirectly affect the action potential of presynaptic neurons to induce GABA release [109, 110] (Fig. 8B, C).

IL-6 has also been reported to have a regulatory effect on GABAergic neurons, mainly by reducing the density of functional GABA receptor A (GABA_A_R), increasing the probability of GABA_A_R leaving the plasma membrane or decreasing the probability of GABA_A_R insertion into the plasma membrane. Therefore, the effect of GABA is attenuated, and the inhibitory or excitatory balance of postsynaptic neurons is disrupted [111]. The regulatory effect of TNF-α on GABA is also related to its receptor. Studies have shown that TNF-α binds to TNF-R1, activates the MAPK, PI3K, protein phosphatase 1 and dynein GTPase 1 signaling pathways, downregulates the levels of the α1, α2, β2, β3 and γ2 subunits of GABA_A_R on the cell membrane surface, and rapidly and sustainably decreases inhibitory synaptic strength in hippocampal GABAergic neurons of adult rat and mouse [112]. The mixed administration of IL-1β, TNF-α and GM-CSF to mice also showed that proinflammatory cytokines can increase the dysfunction of GABA_A_R by increasing the conversion of the GABA_A_R α1-subunit, accelerating the attenuation of GABA IPSC, and weakening its neuroinhibitory effect [113] (Fig. 8B).

Recent studies have shown that IL-10 can regulate the release of GABA from presynaptic neurons in the amygdala through the MAPK and PI3K pathways or indirectly affect the release of GABA from presynaptic neurons induced by action potentials, thereby alleviating anxiety-like behaviors. One study also revealed that the regulatory effect of IL-10 on GABA release in the amygdala of mice with alcohol addiction was comprehensively weakened, and the level of GABA release in the amygdala of mice with alcohol addiction increased after the remission of alcohol addiction, suggesting that the key mechanism involved in the effect of alcohol addiction is the dysfunction of IL-10 on GABA regulation. Therefore, IL-10 can be used as a diagnostic indicator for alcohol addiction and can be applied in the treatment of alcohol addiction through the regulation of GABA release [30] (Fig. 8C).

#### Glu

**Fig. 9:**
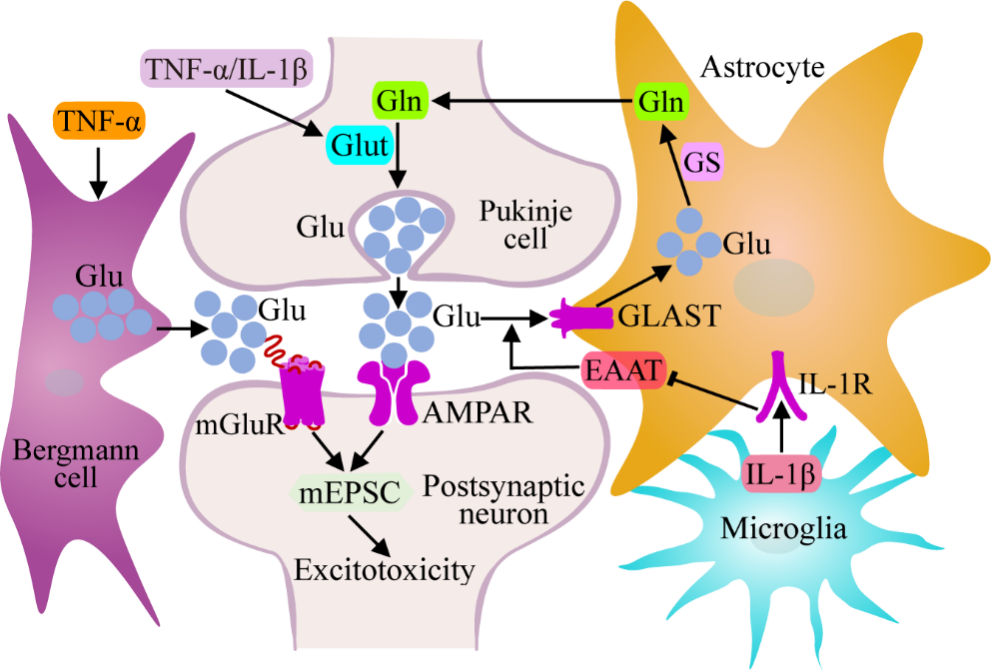
Effects of inflammatory cytokines on Glu synthesis, release and uptake. TNF-α and IL-1β can promote the synthesis and release of Glu, activate mGluR and AMPAR on the postsynaptic membrane, and cause mEPSC enhancement and neuronal excitotoxicity in postsynaptic neurons. Moreover, microglia-derived IL-1β can inhibit the expression of EAAT, reduce the transfer of Glu from the synaptic cleft to astrocytes, and aggravate the hyperexcited stimulation of postsynaptic neurons by Glu. In addition, astrocytes absorb less Glu, transform and generate less Gln, and transport less Gln to glutamatergic neurons, a process that would balance Glu release from glutamatergic neurons to a certain extent

Glu, also known as alpha-amino glutaric acid, is the most abundant amino acid neurotransmitter in the central nervous system. As the precursor of GABA production in the brain, Glu release causes the excitation of postsynaptic neurons, and together with GABA, Glu regulates the balance between excitation and inhibition in the brain [114]. Glu in neurons can be produced by glucose bypass metabolism through tricarboxylic acid (TCA) cycle. After release, Glu is ingested by excitatory amino acid transporters (EAATs) of astrocytes and synthesized into Gln for storage. Gln from astrocytes is then transported to neurons, where it is metabolized back into Glu in response to Glut (Figs. 8A and 9). Insufficient Glu leads to decreased excitability of neurons, and excessive release of Glu leads to hyperexcitability poisoning of postsynaptic neurons [114]. Abnormal Glu synthesis and release are believed to be related to memory disorders, cognitive impairment, schizophrenia, depression, epilepsy and other neurological and psychiatric diseases [79].

Studies on the regulation and mechanism of inflammatory cytokines in glutamatergic neurons have focused mainly on the effect of inflammatory cytokines on Glu uptake by glial cells. For example, activated microglia release large amounts of IL-1β, which can lead to a decrease in EAAT activity and Glu uptake in astrocytes. The excessive presence of glutamate in the synaptic cleft leads to sustained activation of Ca^2+^-permeable α-amino-3-hydroxy-5-methyl-4-isoxazole-propionicacid receptor (AMPAR) in postsynaptic neurons, resulting in hyperexcitability [115, 116] (Fig. 9). Similar results have been demonstrated in mouse models of multiple sclerosis (MS), where IL-1β decreases EAAT expression in cerebellar astrocytes and enhances the effect of Glu in Purkinje cells. The duration of the miniature excitatory postsynaptic currents (mEPSCs) is increased, resulting in hyperexcitability poisoning of postsynaptic neurons [117] (Fig. 9). However, in the treatment of glaucomatous retinal detachment, IL-1 plays a positive regulatory role in Glu uptake by glial cells. The binding of IL-1 to IL-1R in Muller cells can activate the P38 MAPK pathway and increase the expression of caspase 11, which can promote actin fiber depolymerization mediated by cofilin. As a result, actin-loaded Na^+^/K^+^-ATPase is more localized to the cell membrane, and the Na^+^ in Muller cells is transported to the outside of the cell. However, the glutamate transporter (GLAST) on the cell membrane needs to transfer three Na^+^ ions in coordination with each Glu ingested, so Na^+^/K^+^-ATPase increases the efficiency of Glu ingestion through GLAST [118].

In addition, Shim et al. treated normal and TNFR1-shRNA-transfected Bergmann glial cells with TNF-α and found that TNF-α promoted the release of Glu from Bergmann cells to the synaptic cleft of Purkinje cells. It also enhanced the excitability of postsynaptic neurons by activating metabotropic glutamate receptor 1 (mGluR1) [119] (Fig. 9). It has also been shown that inflammatory cytokines can directly regulate the synthesis and release of Glu by neurons. When exploring the neurotoxicity of TNF-α and IL-1β, Ye et al. reported that both could lead to simultaneous increases in intracellular and extracellular Glu content in human cortical neuron cells supplemented with Gln in culture medium, and Glut was no longer localized only in the mitochondrial membrane, but also in other intracellular or extracellular regions. These results indicate that TNF-α and IL-1β can cause neuronal excitatory intoxication by promoting the synthesis and release of Glu [120] (Fig. 9). In contrast, Zhou et al. reported that IL-1β can upregulate the degradation of the synaptophysin protein by the E3 ligase siah1 via the ERK signaling pathway and subsequently inhibit the release of Glu, which impaired the learning and memory ability of rats [121].

### BDNF deficiency and dysfunction

BDNF typically exists as a dimer protein, with each monomer being a secreted, mature polypeptide composed of 119 amino acid residues. BDNF is predominantly expressed in the central nervous system and exerts significant neurotrophic effects. It is the most abundant neurotrophic factor in the adult brain, particularly in the hippocampus and cerebral cortex, where it plays crucial roles in axon growth, myelination, synaptogenesis, and synaptic plasticity [122–124]. Compared to other neurotrophic factors, BDNF has garnered considerable attention in depression research due to its involvement in neuronal repair during depression remission [124–127]. BDNF’s significance in this context has led to a focus on its mechanisms of action and interactions with inflammatory factors, which can impact neural activities through various neurotrophic pathways. Given its importance, BDNF will be used as a representative neurotrophic factor to discuss the effects and mechanisms of inflammatory factors on neural activities mediated by neurotrophic factors. Blood samples and depression scale scores from elderly patients with depression show that the levels of TNF-α and BDNF are negatively correlated, and the ratio of TNF-α to BDNF is consistent with the Montgomery-Asberg Depression Rating Scale (MADRS) [128]. Other studies have confirmed that TNF-α, IL-1β and IL-6 reduce the expression of BDNF in the brain [129, 130], which further attenuates the activation of PI3K/Akt and MAPK P38, downstream signaling molecules of TrkB (transmembrane receptor of BDNF on neurons), leading to structural damage and dysfunction of neurons [26, 131, 132]. These results suggest that inflammatory cytokines can induce BDNF deficiency and dysfunction.

Furthermore, BDNF/TrkB signaling often occurs at the end of axons, and BDNF and TrkB can first form endosomes through endocytosis and then undergo retrograde transport to the cell body, leading to ERK5 phosphorylation and subsequent downstream effects. Carlos et al. cultured the cell body and axon compartments of primary neurons to explore the mechanism by which IL-1β affects BDNF and found that IL-1β attenuated the density of BDNF endosomes in various regions of neurons and that BDNF endosomes accumulated only in the terminal region of axons. Subsequent investigations confirmed that IL-1β induces the ubiquitination of BDNF endosomes, and these ubiquitinated endosomes are discarded after sorting by the cell transport system, and the failure of retrograde transport blocked the downstream signal activation of BDNF [133, 134]. In addition, in a study showing that IL-1β affects long-term potentiation (LTP) in the dendritic spines of hippocampal neurons, IL-1β inhibited the phosphorylation of insulin receptor substrate 1, a protein that links the activation of the BDNF receptor TrkB to downstream signaling pathways regulating CREB, Arc and cofilin, thereby blocking the effect of BDNF, attenuating P38 MAPK activation and actin fiber polymerization, and interfering with memory consolidation [135].

### Neuroplasticity attenuation

Neuroplasticity mainly refers to the regulation and rearrangement of neuronal genesis, differentiation and apoptosis, among which the plasticity of neuronal synapses is the most commonly studied target in disease development and repair. Synaptic plasticity mainly includes short-term synaptic plasticity and long-term synaptic plasticity. Short-term synaptic plasticity includes facilitation, inhibition and enhancement. Long-term synaptic plasticity is primarily manifested as LTP and long-term depression (LTD). In menopause model mice subjected to bilateral ovariectomy, neuronal plasticity in the hippocampus, hypothalamus and amygdala was altered, synaptic transmission and LTP of neurons were significantly weakened, and mice exhibited obvious spatial memory deficits and depressive behaviors [136].

Inflammatory cytokines levels during menopause play important roles in regulating synaptic plasticity. It has been reported that a high concentration of TNF (1 µg/mL) can impair the plasticity of hippocampal CA1 pyramidal neurons in mice without affecting baseline synaptic transmission or previously established LTP. In contrast, a low concentration of TNF (1 ng/mL) promotes LTP in neurons, and the release of intracellular Ca^2+^ can prevent the negative effect of a high concentration of TNF on synaptic plasticity, but when Ca^2+^ stores are depleted, the ability of a low concentration of TNF to promote synaptic plasticity is lost [137]. Recent studies have shown that the MAPK signaling pathway may mediate the inhibitory effect of TNF-α on hippocampal LTP [138].

IL-6 also interferes with synaptic plasticity. The paired pulse current (presynaptic and postsynaptic) in mPFC neurons was measured by the patch clamp technique in IL-6 signal-blocking and wild-type social failure chronic stress model mice. The ratio of current generated by N-methyl-D-aspartate receptor (NMDAR) and AMPAR in stress-tolerant wild-type mice was significantly lower than that in IL-6 signal-blocking mice, and the ratio of current generated by AMPA and GABA_A_R in stress-sensitive wild-type mice was also significantly lower than that in IL-6 signal-blocking mice, indicating that IL-6 drives the negative rearrangement of synaptic plasticity in mPFC neurons during the formation of social failure behaviors [139]. IL-6 has also been shown to play a key role in synaptic plasticity dysfunction in amygdala neurons in mice with peripheral nerve injury-induced depressive behavior. IL-6 activates the palmitoylated transferase DHHC3 through the PI3K signaling pathway and promotes the palmitoylation of postsynaptic density protein-95 (PSD-95), which binds to the postsynaptic membrane Glu receptor 1 and aspartate receptor subunit NR2B, impinging on amygdala neurons from the rest of the brain, resulting in depression-like behavior in mice [140]. Tocilizumab, an IL-6 receptor antagonist, can inhibit the activation of hippocampal microglia and astrocytes and the subsequent “inflammatory storm” through the Wnt/β-catenin signaling pathway and restore hippocampal synaptic plasticity [141].

Additionally, INF-γ is involved in the regulation of neuroplasticity, which can lead to synaptic structural instability by reshaping the morphology of neurons in the nucleus accumbens, thus affecting opioid-induced addictive withdrawal behavior [142]. INF-γ can induce an acute increase in the amplitude of IPSCs in GABAergic neurons in a postsynaptic protein kinase C (PKC)-dependent manner and an acute increase in the frequency of IPSCs in GABAergic neurons in an inducible nitric oxide synthase- and soluble guanylate cyclase-dependent manner. IFN-γ was observed to shift short-term synaptic plasticity toward facilitation [143].

The immune factors IL-1β, IL-10 and IL-33 also play roles in the regulation of neuroplasticity. IL-1β inhibits the expression of the synaptic formation-related molecules synaptophysin (SYP and SYN1), the postsynaptic protein α-amino-3-hydroxy-5-methyl-4-isoxazolepropionic acid receptor GluA1 subunit, the N-methyl-d-aspartate receptor NR2B subunit and PSD-95 through histone deacetylase 4 (HDAC4), thus regulating the transcriptional inhibitor MeCP2 SUMO, resulting in LTP deficits and decreased synaptic plasticity [144].

IL-10 has been found to significantly enhance synaptic transmission and synaptic plasticity in hippocampal glutamatergic neurons, increasing the frequency of mEPSCs in a dose-dependent manner. In addition, IL-10 can induce postsynaptic compensatory changes such as synaptic expansion, increased mEPSC amplitude, and enhanced Ca^2+^ responsiveness to the AMPA agonist 5-fluorouracil alanine in primary cultured hippocampal glial synapses [145].

The regulation of neuroplasticity by IL-33 was discovered in the study of the mechanism of memory consolidation. Both memory accuracy and the expression of IL-33 decreased simultaneously in aged mice, and IL-33 administration significantly alleviated age-related dendritic spine regression. IL-33 can also guide microglia to phagocytose the extracellular matrix, which is conducive to the remodeling of synaptic connections between neurons and the functional integration of new neurons and increases the accuracy of fear memory [146]. As a new member of the IL-1 family, IL-33 is considered to play a role similar to that of a warning activator in the positive regulation of synaptic plasticity; however, the detailed mechanism is still unclear.

## Therapeutic drugs for menopausal depression that target estrogen-immuno-neuromodulation

Currently, the primary therapeutic drugs used to treat menopausal depression include fluoxetine, duloxetine, escitalopram, mirtazapine, desvenlafaxine, quetiapine, modafinil and other neuromodulators [147–153]. However, these drugs do not address the decline in ovarian function and estrogen levels in menopausal women, so their therapeutic effect on menopausal depression is not ideal. Consequently, ERT has gained attention as a potential treatment. E2, ERβ selective ligand C-1, Vivelle-Dot, G03C, G03F, E2 + progesterone and tibolone have been used to treat model animals or menopausal depression patients [154–159]. Additionally, immune imbalances in menopausal women are also of interest to researchers, and immunomodulatory drugs and agents, such as RSV, SIM, astragalin, Erxian decoction, omega-3 (ω-3, also called N-3) polyunsaturated fatty acid, *Fagopyrum tataricum* seed extract, *Acer tegmentosum*, Saikosaponin A and Chaihu-Guizhi-Ganjiang tang, have been used to treat menopausal depression model animals or perimenopausal and postmenopausal women with mood disorders and shown good curative efficacy. However, the targets of most of these drugs and agents remain unclear [25, 43–45, 67, 160–164]. Here, we summarize the names, known targets, subjects and efficacy of these therapeutic drugs and agents to provide a reference for further evaluation, improvement or clinical trial research (Table 1).

**Table 1:** Therapeutic drugs/agents for menopausal depression targeting the estrogen-immune-neuromodulation signaling pathway

Drugs/Agents	Targets		Subjects	Efficacy
E2	ERα, ERβ		Ovariectomized rats	Synergized with escitalopram to exert anti-menopausal depression effects [154]
ER-β selective ligand C-1	ERβ		Ovariectomized mice	Reduced depressive-like behavior and also avoided uterine obesity in ovariectomized mice [155]
Vivelle-Dot	ER		Women with past perimenopausal depression and women with no history of depression	Reduced the recurrence and incidence of depression [156]
G03C, G03F	ER, PR		Women in Denmark aged 45 years and over	Decreased the risk of depression for women aged 54 or older when were administered locally [157]
E2 + progesterone	ER, PR		Euthymic perimenopausal and early postmenopausal women	Effectively prevented the development of clinically significant depressive symptoms [158]
Tibolone	ER, PR, AR		Perimenopausal women with depressive symptoms	Exhibited exciting innovations for the treatment of menopausal depression through tissue-selective activation of ER, PR or AR [159]
RSV	SIRT1		Ovariectomized mice	Ameliorated depression- and anxiety-like behaviors by inhibiting the activation of NLRP3 and NF-κB in the hippocampus [43]
SIM	Hydroxymethylglutaryl coenzyme A reductase		Ovariectomized rats	Alleviated depressive behavior in ovariectomized rats by inhibiting the activation of microglia and NLRP3 inflammasome, and the expression of P2X7R, TLR2 and TLR4 [44]
Astragalin	Unclear	Ovariectomized mice		Attenuated depression‑like behaviors by regulating the IL‑4R/JAK1/STAT6 signaling pathway [25]
Erxian decoction	Unclear	Ovariectomized mice		Relieved perimenopausal depression by regulating the levels of BDNF, Bcl-2, ER and IL-6 [160]
ω-3 polyunsaturated fatty acid	Unclear	Ovariectomized rats		Exerted antidepressant and neuroprotective activities through suppressing microglial M1 polarization and inhibiting proinflammatory cytokines expression [45]
N-3 polyunsaturated fatty acid	Unclear		Ovariectomized rats	Decreased the levels of IL-6 and TNF-α and improved depressive behaviors in postmenopausal depression rats [161]
*Fagopyrum tataricum* seed extract	Unclear		Ovariectomized rats	Inhibited the occurrence of depression-like behavior in ovariectomized rats by decreasing the expression of IL-1β and IL-2 [67]
*Acer tegmentosum*	Unclear		Ovariectomized rats	Effectively decreased behavioral depression-like responses by downregulating the expression of c-Fos and IL-1β [162]
Saikosaponin A	Unclear		36-week-old rats with chronic unpredictable mild stress	Exerted the antidepressant-like effects by regulating the levels of IL-1β, IL-6 and TNF-α in the hippocampus [163]
Chaihu-Guizhi-Ganjiang tang	Unclear		Perimenopausal and postmenopausal women with mood disorder	Reduced plasma IL-6 and soluble IL-6 receptor concentrations and improved depressed mood [164]

PR, progesterone receptor; AR, androgenic receptor

## Conclusion and future directions

**Fig. 10:**
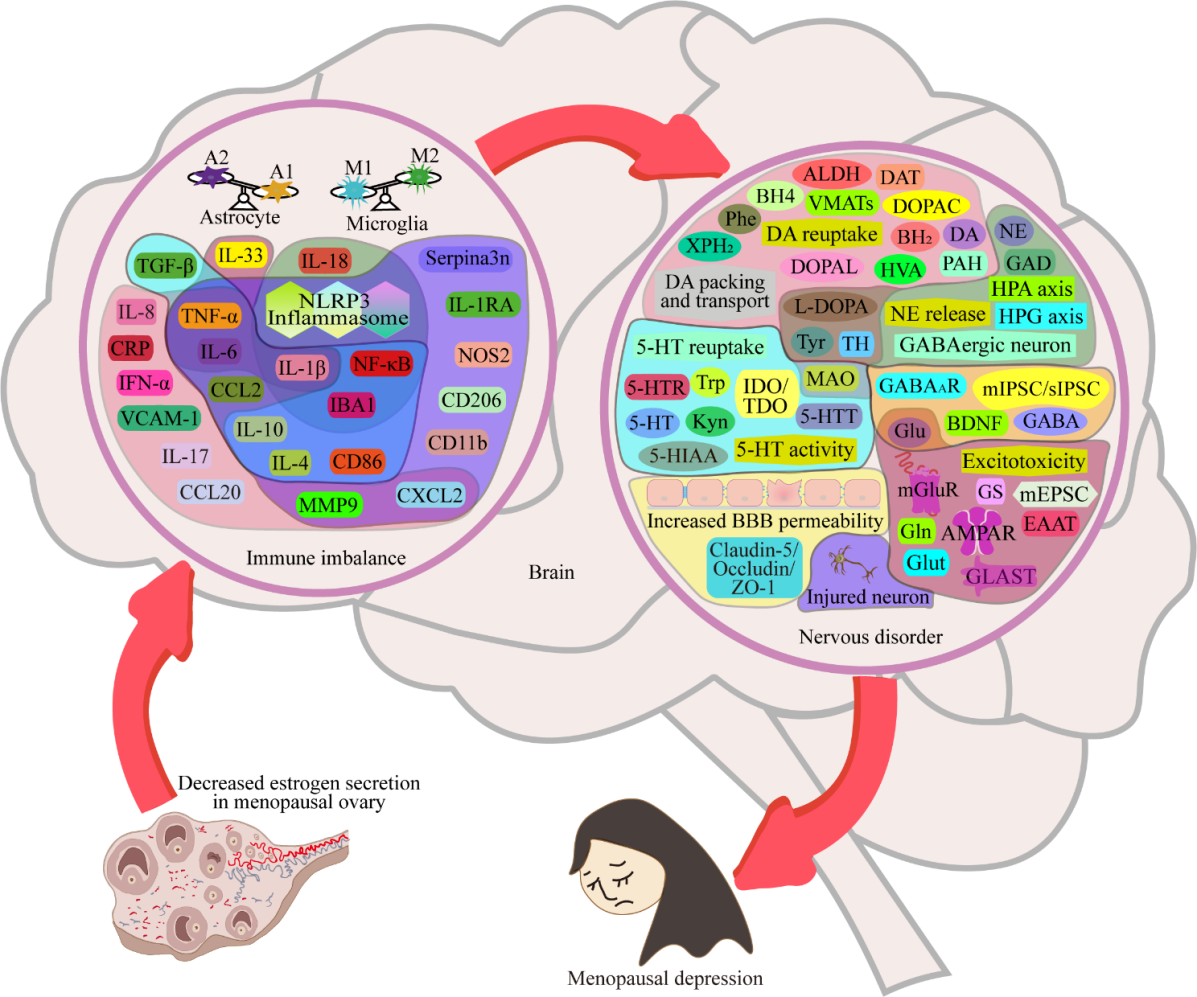
Estrogen-immune-neuromodulation disorders promote menopausal depression. A significant decrease in estrogen levels in menopausal women disrupts the homeostasis of the estrogen-immune-neuromodulation system, which first leads to immune imbalance, then induces nervous disorders, and finally gradually causes depression in menopausal women. Current research on immune imbalance has focused mainly on microglia, astrocytes, proinflammatory factors such as TNF-α, IL-1β, and IL-6, and anti-inflammatory factors such as IL-4 and IL-10. The investigation of neurological impairments involves BBB permeability, neurotransmitter activity, BDNF synthesis and neuronal plasticity. All these advances provide clues for the targeted treatment of menopausal depression

In conclusion, a significant decrease in estrogen levels disrupts immune homeostasis, particularly the levels of the immune factors TNF-α, IL-1β, IL-4, IL-6, IL-10 and IL-18 through the ERα/ERβ/GPER-associated NLRP3/NF-κB signaling pathway, which involves many signaling molecules, such as TLR2, TLR4, P2X7R, SIRT1, CREB, MS-KIF18A, PI3K, AKT, Gs, cAMP, PKA, PJA1, Serpina3n, Src, and MMP-9 [19–24, 41, 43, 44, 47–49, 52, 54–56, 58]. Subsequently, the increase of inflammatory cytokine levels causes BBB destruction [25, 62–74, 165], the dysfunction of neurotransmitters such as 5-HT [80–85], DA [86–97], NE [98–105], GABA [29, 30, 106–113] and Glu [79, 114–121], BDNF deficiency and dysfunction [26, 128–135], and attenuation of neuroplasticity [136–146], which are key factors in the onset and progression of depression (Fig. 10). All of these studies have shown that there is a complex regulatory system from estrogen to immune signaling molecules to neurons, which we call the estrogen-immune-neuromodulation system, and estrogen-immune-neuromodulation disorders cause susceptibility to depression in menopausal women (Fig. 10). Therefore, drugs targeting inflammatory cytokines and NLRP3/NF-κB signaling pathway-associated molecules are promising for restoring homeostasis of the estrogen-immuno-neuromodulation system and may play a positive role in the intervention and treatment of menopausal depression.

Although the main framework of the estrogen-immune-neuromodulation system is understood, many details involving this regulatory system remain unclear. For example, studies on the downstream signaling molecules of ERs and the stimulating factors of the NLRP3/NF-κB signaling pathway are very extensive, and few studies on the activation of NLRP3 and NF-κB caused by estrogen deficiency have clarified the interactions between various molecules step by step, which has limited the development of targeted antidepressants. In addition, there are some opposing and unified equilibrium points in the estrogen-immune-neuromodulation system. Multiple studies focusing on IL-1β have shown that IL-1β can not only decrease NE synthesis by disrupting the HPG system, GABA neurons and the expression of TH but also promote an increase in NE synthesis through the HPA system [98, 99, 103] (Fig. 7). IL-1β acts on different GABA neuron clusters in the amygdala and shows positive and negative regulatory effects [108–110] (Fig. 8B). Coincidentally, low levels of IL-33 and other new members of the IL-1 family show a positive regulatory effect on neuroplasticity, while high levels of IL-33 lead to a decrease in BDNF synthesis [29, 107] (Fig. 8A), disrupting the construction of neural synapses. Our understanding is that under normal circumstances, different elements of the body control a physiological activity in an appropriate state through mutual restriction and coordination, so different doses of stimulus factors affected by time and space are critical to maintaining or upsetting the balance. The dramatic decrease in estrogen levels in menopausal women triggers an imbalance in the estrogen-immune-neuromodulation system, which is the underlying reason why menopausal women are more susceptible to depression. Therefore, accurately understanding the appropriate balance in the nervous system and the regulatory mechanism is key for improving the clinical treatment of menopausal depression.

### Electronic supplementary material

Below is the link to the electronic supplementary material.

Supplementary Material 1

## Data Availability

No datasets were generated or analysed during the current study.
