# Supplementary material for: Estrogen-immuno-neuromodulation disorders in menopausal depression
Source: J Neuroinflammation. 2024 Jun 19;21:159. doi: 10.1186/s12974-024-03152-1 (PMC11188190; doi:10.1186/s12974-024-03152-1)
Supplement: Supplementary file 1 — Supplementary Material 1 [file 12974_2024_3152_MOESM1_ESM.doc]

**Abbreviations**

| 5-HIAA | 5-hydroxyindoleacetic acid | Kyn | kynurenine |
| --- | --- | --- | --- |
| 5-HT | 5-hydroxytryptamine | L-DOPA | levodopa |
| 5-HTR | 5-HT receptor | LTD | long-term depression |
| 5-HTT/SERT | 5-HT transporter | LTP | long-term potentiation |
| ALDH | aldehyde dehydrogenase | MAO | monoamine oxidase |
| AMPAR | α-amino-3-hydroxy-5-methyl-4-isoxazole-propionicacid receptor | MAPK | mitogen-activated protein kinase |
| Arc | arcuate nucleus | MCP | macrophage cationic peptide |
| Arg | arginase | mEPSC | miniature excitatory postsynaptic current |
| BBB | blood-brain barrier | mGluR | metabotropic glutamate receptor |
| BDNF | brain-derived neurotrophic factor | MIP | macrophage inflammatory protein |
| BH_2_ | dihydrobiopterin | mIPSC | miniature inhibitory postsynaptic current |
| BH_4_ | tetrahydrobiopterin | MMP | matrix metalloproteinase |
| cAMP | cyclic adenosine monophosphate | MPA | medial preoptic area |
| CCL | C-C motif chemokine ligand | mPFC | medial prefrontal cortex |
| CeA | central amygdala | MS-KIF18A | marrow stromal kinesin family member 18A |
| CRP | C-reactive protein | NE | norepinephrine |
| CSF | cerebrospinal fluid | NF-κB | nuclear factor kappa-B |
| CXCL | CXC-chemokine ligand | NLRP | nucleotide-binding oligomerization domain-like receptor protein |
| DA | dopamine | NOS | nitric oxide synthase |
| DAT | DA transporter | P2X7R | purinergic ligand-gated ion channel 7 receptor |
| DOPAC | 3, 4-dihydroxyphenylacetic acid | PAH | phenylalanine hydroxylase |
| DOPAL | 3, 4-dihydroxyphenzene acetaldehyde | p-CREB | phosphorylated cAMP-responsive element binding protein |
| E2 | estradiol | p-ERK | phosphorylated extracellular signal regulated protein kinase |
| EAAT | excitatory amino acid transporter | Phe | phenylalanine |
| ER | estrogen receptor | PJA-1 | Praja-1 |
| ERT | estrogen replacement therapy | PKA | protein kinase A |
| GABA | γ-aminobutyric acid | p-PKB/AKT | phosphorylated protein kinase B |
| GABA_A_R | GABA receptor A | ROS | reactive oxide species |
| GAD | glutamic acid decarboxylase | RSV | resveratrol |
| GLAST | glutamate/aspartate transporter | SIM | simvastatin |
| Gln | glutamine | sIPSCs | spontaneous inhibitory postsynaptic currents |
| Glu | glutamate | SIRT1 | silent information regulator factor 2-related enzyme 1 |
| Glut | glutaminase | SSRI | selective serotonin reuptake inhibitor |
| GPER | G protein-coupled estrogen receptor | TCA | tricarboxylic acid |
| GPR | G protein coupled receptor | TDO | tryptophan 2, 3-dioxygenase |
| Gs | stimulating adenylate cyclase G protein | TH | tyrosine hydroxylase |
| GS | glutamine synthetase | TLR | toll-like receptor |
| HPA | hypothalamic pituitary adrenal | TNF | tumor necrosis factor |
| HPG | hypothalamic pituitary gonadal | TrkB | tropomyosin receptor kinase B |
| HVA | homovanillic acid | Trp | tryptophan |
| IBA1 | ionized calcium-binding adapter molecule 1 | Tyr | tyrosine |
| IDO | indoleamine 2, 3-dioxygenase | VCAM | vascular cell adhesion molecule |
| IFN | interferon | VMAT | vesicle monoamine transporter |
| IL | interleukin | XPH_2_ | dihydroxanthopterin |
| IL-1RA | IL-1 receptor antagonist | ZO-1 | zona occludens-1 |
